# Efficacy, Safety, and Cost-Effectiveness of Healthium Theruptor Versus 3M Tegaderm Versus Plain Gauze Dressing for Wound Dressings Used in Abdominal and Joint Surgeries: A Prospective, Multicentric, Randomized Study

**DOI:** 10.7759/cureus.53947

**Published:** 2024-02-10

**Authors:** Michael Rodrigues, Shoban Varthya, Vinoth Sunderasan, Tharun Ganapathy, Sakthi Balan, Gayathri Sivakumar, Mayank Badkur, Meenakshi Gothwal, Sneha Ambwani, Jaykaran Charan, Uthpala Vadakaluru, Ashok Kumar Moharana, Deepak Siddabasavaiah

**Affiliations:** 1 Research & Development (CareNow Medical Private Limited), Healthium Medtech Limited, Bengaluru, IND; 2 Pharmacology, All India Institute of Medical Sciences, Jodhpur, Jodhpur, IND; 3 General Surgery, Mahatma Gandhi Medical College and Research Institute, Puducherry, IND; 4 General Surgery, Sri Ramaswamy Memorial (SRM) Institute of Science and Technology, Chennai, IND; 5 Clinical Research, Ki3 Pvt. Ltd., Puducherry, IND; 6 General Surgery, All India Institute of Medical Sciences, Jodhpur, Jodhpur, IND; 7 Clinical Affairs, Healthium Medtech Limited, Bengaluru, IND

**Keywords:** wound healing, theruptor, tegaderm, surgical site infections, plain gauze

## Abstract

Background

In the realm of surgical and postoperative care, the application of wound dressings is a standard practice to facilitate healing, minimize infection risks, and offer a protective barrier against pathogens for optimal recovery. For instance, Theruptor is an active advanced wound care product with patented microbicidal technology. In the present study, we conducted a randomized clinical trial to compare the clinical efficacy and safety of Healthium Theruptor, 3M Tegaderm, and plain gauze dressings in patients undergoing abdominal and joint surgeries.

Methodology

This was a multicenter, prospective, three-arm, randomized, double-blind study conducted between April and November 2022 at three different sites in India, viz., All India Institute of Medical Sciences, Jodhpur; Mahatma Gandhi Medical College and Research Institute, Puducherry; and SRM Institute of Science and Technology, Chennai. A total of 210 patients were randomized to receive either of the following three interventions: Theruptor, Tegaderm, and plain gauze dressing (n = 70 each) based on computer-generated randomization sequences using sequentially numbered, opaque, sealed envelopes. Demographic data and surgery details were obtained and recorded at baseline. Parameters such as rate of wound healing, incidence of surgical site infections (SSIs), adverse events, product performance, and pain score were assessed and compared during the weekly follow-up visits until 28 days. In addition, wound assessments using the Stony Brook Scar evaluation scale, Cardiff Wound Impact Questionnaire, and Modified Hollander Wound Evaluation Scale were conducted to provide additional insights on the efficacy of the dressings (days 3, 7, 14, and 28). Lastly, the cost of wound management was assessed at the end of the study. The statistical analysis of the data was performed using a one-way analysis of variance followed by a Bonferroni post-hoc test on GraphPad software.

Results

All three dressings were equally effective in healing the wound and reducing the incidence of SSIs. The median healing time was estimated to be seven days. Further, no significant difference was observed in wound dehiscence, wound pain, clinical wound parameters, cosmetic assessment, and quality of life among the three groups (p > 0.05) during the follow-up visits. However, the product performance of Theruptor and Tegaderm was significantly better than plain gauze dressing in terms of ease of application (82.87% and 84.13% vs. 71.7%), ease of removal (83.09% and 83.67% vs. 70.79%), comfort to wear (82.59% and 84.47% vs. 72.83%), exudate management (84.35% and 85.7% vs. 77.23%), mean wear time in hours (65.57 and 65.92 vs. 49 hours), and mobility of the patient (p < 0.05). Further, the total cost of wound management with Theruptor dressing was significantly lower than with Tegaderm dressing (₹1117.2 ± 269.86 vs. ₹1474 ± 455.63; p < 0.0001).

Conclusions

Although all three dressings were equally safe and clinically efficacious, Theruptor was more cost-effective with better product performance. Thus, Theruptor may be a considerate option in the postoperative wound management of abdominal and joint surgeries.

## Introduction

Healthcare-associated infections (HAIs) are those that are acquired in hospital settings while receiving care services and health treatments. HAIs are considered a major public health concern associated with high mortality [1]. The World Health Organization and Centers for Disease Control and Prevention reports revealed that approximately 1.7 million hospitalized patients acquire HAIs every year and about 98,000 of these patients, i.e., one in every 10 affected patients, die from HAIs [1-3].

HAIs are of different types such as central line-associated bloodstream infections, surgical site infections (SSIs), catheter-associated urinary tract infections, and ventilator-associated pneumonia [1,4]. Among them, SSIs, previously known as wound infections, are the most common adverse events (AEs) associated with invasive surgical procedures [1,5]. Regardless of advances in preventive procedures, SSIs cause approximately 15% of complications after surgery [6]. Evidence in the literature suggests that orthopedic surgeries followed by cardiac and intra-abdominal surgery are at the highest risk of developing SSIs with a prevalence of 2-36% [6,7]. In a Japanese study, Watanabe et al. (2008) conducted SSI surveillance in 27 hospitals and estimated the overall SSI rate in abdominal surgeries as 15.5% [8]. The risk of developing SSIs can be avoided and prevented through several methods such as skin antisepsis, surgical hand hygiene technique, antibiotic prophylaxis, maintaining normothermia, using wound protectors, increasing oxygen delivery, hair removal, and use of sterile dressings [9].

Currently, there is a wide variety of commercially available polymeric wound dressings that help prevent SSI and enhance wound closure and healing [10]. These dressings are designed to provide a suitable environment for wound healing, reduce the risk of infections, and enhance the overall healing process. For example, polyurethane, hydrocolloids, hydrogels, films, foams, and composite dressings. Based on the characteristics of the wound and healing stage, a specific type of dressing is chosen [10]. The range of treatment can extend, on one hand, from general practice where a plain gauze dressing has commonly been used to treat wounds since ancient times. In certain cases, it is supported by an elasticated crepe bandage to exert pressure to stop bleeding, absorb exudates, and promote healing [10]. On the other hand, Tegaderm (3M) is an old commonly used wound dressing composed of a polyurethane film, silicone-coated paper liner, and a non-adherent pad with acrylate adhesive [11]. Substantial studies in the literature have reported that Tegaderm has a unique combination of properties, including comfort, prevention of further trauma, moisture, exudate wicking, cost-effective, water-proof, easy to remove, and antimicrobial properties [12,13]. Tegaderm and plain gauze dressings are frequently used dressings in clinical settings. Recently, an active advanced wound care product, Theruptor (Healthium Medtech Limited, India), with patented microbicidal technology and several other significant benefits has emerged. It consists of a three-dimensional (3D) knitted hydrocellular textile substrate made of 90% w/w polyethylene terephthalate and 10% w/w polyurethane. The 3D substrate is permanently bound and cross polymerized-cross linked with a cationic surfactant called dimethyl tetradecyl [3-(trimethoxy silyl) propyl] ammonium chloride (DTAC). DTAC technology uses a physical kill mechanism for microbial protection. It is immobilized on the substrate and does not leach out of the dressing [14]. However, the comparative data on the efficacy of these dressings is limited. To fill a crucial gap in our understanding of the comparative effectiveness of these wound care interventions, we conducted a multicentric, prospective, randomized, double-blind study to assess and compare the clinical efficacy and safety of Healthium Theruptor, 3M Tegaderm, and plain gauze dressing in patients undergoing abdominal and joint surgeries.

## Materials and methods

Trial design

This was a multicentric, prospective, three-arm, parallel-group, randomized (1:1:1), double-blind study to compare the clinical efficacy and safety of three wound dressings, i.e., Healthium Theruptor, 3M Tegaderm, and plain gauze dressing. The study was conducted from April 2022 to November 2022.

Ethics

The study was conducted at three different sites in India. The ethical approvals were obtained from their respective institutional review boards. Necessary approvals for the study were also obtained from the Central Drugs Standard Control Organization, the apex body in India giving permissions for clinical Investigations. The study was conducted and reported in compliance with the principles of Good Clinical Practice guidelines and the Declaration of Helsinki. The study was registered in the Clinical Trials Registry - India (registration number: CTRI/2022/03/040898; registered on: 08/03/2022). Written informed consent was obtained from each patient before their enrolment in the study. The clinical trial was reported in accordance with the 2010 Consolidated Standards of Reporting Trials (CONSORT) guidelines [15].

Participants

Based on the inclusion and exclusion criteria, patients undergoing abdominal and joint surgery were enrolled at three different sites in India. The inclusion criteria were male or female patients aged 18 years and above, who underwent emergency/elective abdominal surgery, elective cesarean section, or elective joint surgery, and were willing to provide consent to participate in the study procedures and follow-up evaluations. Patients with the following conditions were excluded: patients with severe anemia, severe malnutrition, serum creatinine of more than 2 mg/dL, HbA1c of more than 10%, primary peritonitis, liver cirrhosis, allergic to interventions, who were on immune system modulators, and who had wounds in which incision edges were under high tension or not easily apposed and hemostasis or deep closure could not be achieved.

Interventions

The patients were randomized into three groups to receive either of the three products, i.e., Healthium Theruptor, 3M Tegaderm, or plain gauze dressing for the wound management and healing of abdominal and joint surgeries. In all three groups, the dressings were applied immediately after skin closure at the end of the surgery. Before applying the dressing material, only normal saline was used to clean the area. Topical antibiotics were not used during the study period. The respective allocated dressings were changed by the nursing staff and replaced by fresh dressings twice a week.

Objectives

At baseline, demographic data and surgery details were obtained and recorded (day 0).

Primary Objective

The primary objective of the study was to compare the rate of wound healing and closure among the three groups. Further, the effectiveness of wound dressings was assessed by evaluating the incidence of SSI.

Primary Endpoint

In all three groups, the rate of wound healing was calculated by estimating the proportion of patients with completely healed wounds during the follow-up visits (days 3, 7, 14, and 28). Further, the incidence of SSI was assessed by performing microbiological screening of the wound site with a swab test during dressing change (days 3, 7, 10, 14, and 28). The swabs were only taken from patients who had any kind of discharge from the wound site or with the presence of any suspected signs of infection during the dressing change and wound cultures were performed. Briefly, the head of the swab was moved from the center of the wound outward to the edge of the wound. The swab was then processed for the culture on specific media under aerobic conditions at 37°C. After subsequent hours, the cultures were checked for the growth of microbes.

Secondary Objectives

The secondary objectives included the determination and evaluation of acceptability, durability, patient compliance, product performance, and safety parameters of the dressings.

Secondary Endpoints

In all three groups, data including wound dehiscence, pain score, clinical assessment of the wound, complications after surgery (Clavien-Dindo classification [16]), patient satisfaction with the dressing and wound healing, cosmetic assessment (modified Hollander Wound Evaluation Scale [17] and Stony Brook Scar Evaluation Scale (SBSES) [18]), Cardiff Wound Impact Questionnaire [19], overall quality of life, product performance, and cost analysis were assessed during the four weeks of follow-up visits. The AEs, serious AEs, and device incidents were also noted.

Wound dehiscence: Wound dehiscence was assessed in percentage on the day of intervention and during the follow-up visits (days 7, 14, and 28).

Wound pain score: The patients were asked to rate the wound pain on a visual analog scale ranging from 0 (no pain) to 10 (worst pain) on the day of intervention and during the follow-up visits (days 7, 14, and 28).

Clinical assessment of wounds: The wound was clinically assessed for purulent drainage, hemorrhage, swelling, erythema, and scab on days 3 and 7. These parameters were graded as none, scant, moderate, and copious.

Product performance: The performance of the product in terms of ease of dressing application and removal, comfort to wear, discoloration to surrounding skin, pain on dressing application, pain on dressing removal, exudate management, and leakage handling of dressing was analyzed in percentages, provided by the surgeons, nurses, and patients during dressing change. Thereafter, the mean values were calculated. A higher percentage represented “good outcomes” for the following sub-parameters: ease of dressing application and removal, comfort to wear, exudate management, and leakage handling of dressing, whereas a lower percentage represented “good outcomes” for discoloration to surrounding skin and pain on dressing application and removal. In addition, the number of dressing changes required, mean wear time of dressing, and mobility of the patients were also documented. The mobility of the patients was rated as normal, good, satisfied, and not satisfied.

Modified Hollander Wound Evaluation scale: Cosmesis was evaluated using the Modified Hollander Wound Evaluation scale [17] on days 3 and 7. It consists of six items, i.e., step-off borders, contour irregularities, margin separation, edge inversion, excessive distortion, and overall appearance. These items are scored either 0 or 1 for the absence and presence of the incision attribute, respectively. The total score ranges from 0 (best) to 6 (worse).

Stony Brook Scar category: On days 10, 14, and 28, the cosmetic assessment of the wound was carried out using the SBSES [18] among the three groups with points 0 and 1. The SBSES includes width (0: >2 mm and 1: <2 mm), height (0: elevated/ depressed and 1: flat), color (0: darker than the surrounding skin and 1: same color or lighter than the surrounding skin), hatch marks/suture marks (0: present and 1: absent), and overall appearance (0: poor and 1: good). The total mean score of the intervention group was calculated and compared.

Overall quality of life: The patients were asked to rate the overall quality of life on a scale of 0 (not at all satisfied) to 10 (very satisfied).

Cardiff Wound Impact Questionnaire: This questionnaire [19] consists of three domains, i.e., social life (14 items related to stress and experience), well-being (7 items), and physical symptoms and daily living (12 items related to experience). All three domains are scored on a scale of 0 (not at all) to 5 (always). The patients were asked to score the scale on days 3, 7, 14, and 28.

Cost analysis: At the end of the study, the cost of wound management in terms of unit costs of all (dressing) materials used and costs of personnel and consumables involved in wound care were assessed in all three groups. The dressing cost was calculated by the number of dressing changes multiplied by the average unit price of the dressing.

Sample size estimation

The sample size was calculated based on the odds ratio of a reference study, namely, Ravenscroft et al. (2006) comparing the efficacy of Tegaderm [20]. With an odds ratio of 5.8, alpha of 0.05, power of 0.98, P1 of 0.46, and P2 of 0.83, the estimated sample size was 55 per group. Considering 10-20% dropouts, the total number of patients included in the study for three arms was 210 (70 in each group).

Randomization and dressing allocation

Randomization was performed by the use of sequentially numbered, opaque, sealed envelopes with unique randomization subject numbers at a ratio of 1:1:1 to ensure an unbiased and balanced allocation of interventions. A computer-based randomization sequence list was generated for the same. Each randomization number with the allocated treatment information was sealed in a separate envelope, referred to as a code-break envelope. All patients, investigators, and other staff members were blinded toward the intervention groups until the completion of the study.

Statistical analysis

The statistical analysis of the data and graph formation was performed using GraphPad software version 5.1. The categorical data were represented as numbers with percentages and continuous data were represented as mean with standard deviation (SD). Primarily, the normality of the data was verified using the Kolmogorov-Smirnov test. Based on the normal distribution of the data, all three groups were compared using a one-way analysis of variance, followed by the Bonferroni post-hoc test. The p-value of <0.05 was considered statistically significant.

## Results

**Figure 1:**
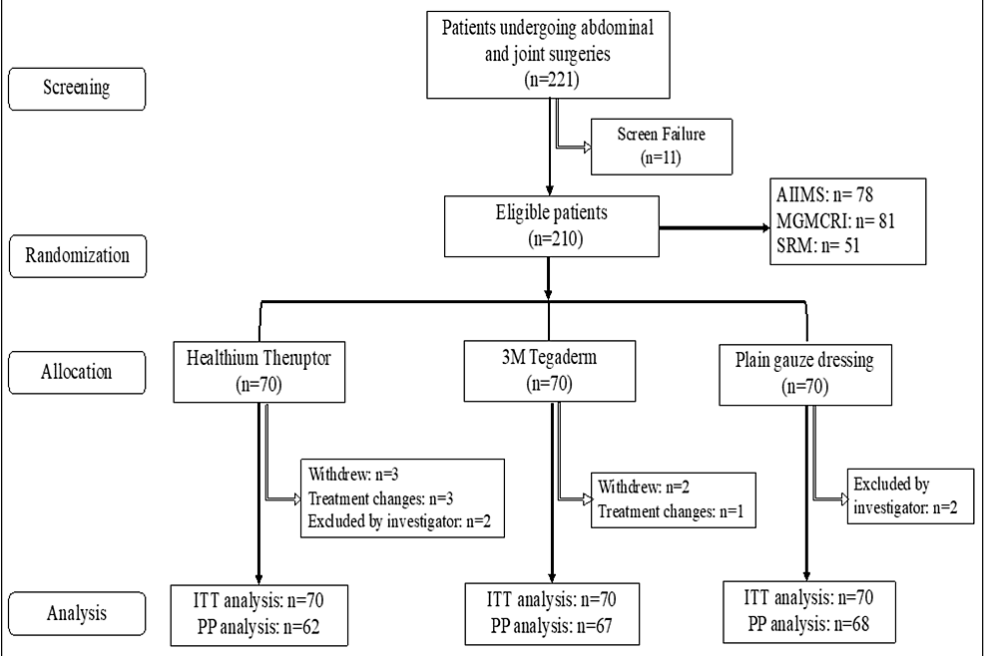
CONSORT flow diagram. n = number of patients; AIIMS= All India Institute of Medical Sciences; MGMCRI = Mahatma Gandhi Medical College and Research Institute; SRM = Sri Ramaswamy Memorial Institute of Medical Sciences; ITT analysis = intention-to-treat; PP = per protocol

A total of 221 patients were screened for recruitment in the study. Among them, 210 patients were subjected to randomization, with 70 patients in each dressing arm. Of the 210 patients, five withdrew from the study, four required conventional treatments, and four were excluded by the investigator. No patient was further lost to follow-up during the study period. The study was conducted at three different sites in India, i.e., Site 1 (n = 78), Site 2 (n = 81), and Site 3 (n = 51), from April 8, 2022, to October 14, 2022. The final follow-up was completed on November 11, 2022. The CONSORT flowchart is shown in Figure 1.

Demographics and diagnosis

The demographic data in all three groups were comparable regarding age, gender, and type of surgery. Among the recruited patients, 79% were females. In total, 98% of patients underwent elective abdominal surgery, and 2% of patients underwent joint surgeries. Table 1 shows details of the demographics and diagnosis of the patients.

**Table 1:** Demographic, medical history, and surgery details of the recruited patients. LSCS = lower (uterine)-segment cesarean section; n = number of subjects; % = percentage; SD = standard deviation

Parameters	Theruptor (n = 70)	Tegaderm (n = 70)	Plain gauze (n = 70)	P-value
Age, years (mean ± SD)	35.8 ± 13.1	34.16 ± 13.8	34.99 ± 13.76	0.739
Sex, n (%)				0.832
Male	13 (18.6)	15 (21.4)	15 (21.4)	
Female	57 (81.4)	55 (78.6)	55 (78.6)	
Type of surgery, n (%)				0.843
Abdominal cases	68 (97.1)	69 (98.6)	69 (98.6)	
Elective LSCS	50 (73.5)	50 (72.5)	47 (68.1)	
Hernioplasty	10 (14.7)	16 (23.5)	13 (18.9)	
Laparotomy	5 (7.4)	1 (1.5)	4 (5.8)	
Others	3 (4.4)	2 (2.5)	5 (7.2)	
Joint cases	2 (2.9)	1 (1.4)	1 (1.4)	

Primary endpoints

Rate of Wound Healing

**Figure 2:**
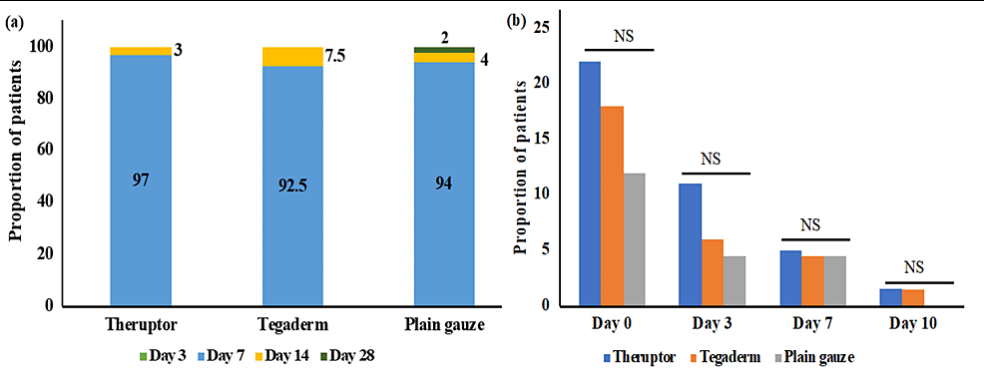
Rate of wound healing and surgical site infections. Bar graphs represent the proportion of patients with (a) wound healing and closure and (b) surgical site infections during the follow-up period of the study. NS: non-significant.

No patients from either of the groups were healed on day 3. The majority of the patients were healed on day 7, i.e., 97% (60/62) of patients belonging to the Theruptor group, 92.5% (62/67) of patients in the Tegaderm group, and 94% (64/68) of patients in the plain gauze dressing group. The proportion of patients with wound healing belonging to the Theruptor group was comparatively higher than the other groups; however, the data were not statistically significant (p > 0.05). The remaining patients from all groups were healed on day 14. Only one patient from the plain gauze group was healed on day 28. The median time for wound closure and healing in all three dressing arms was found to be seven days (Figure 2, Panel a).

Surgical Site Infection

The microbial load was reduced during the follow-up period in all three groups from 17% on day 0 to 1% on day 10. When patients belonging to each group were compared for microbial load (SSI), no significant difference was observed between the groups on the respective days (p > 0.05), suggesting all dressings were equally efficacious in reducing the microbial load (Figure 2, Panel b). Of note, one patient in the Tegaderm group developed complications due to SSI and re-exploration surgery was performed.

Secondary endpoints

Wound Dehiscence

**Figure 3:**
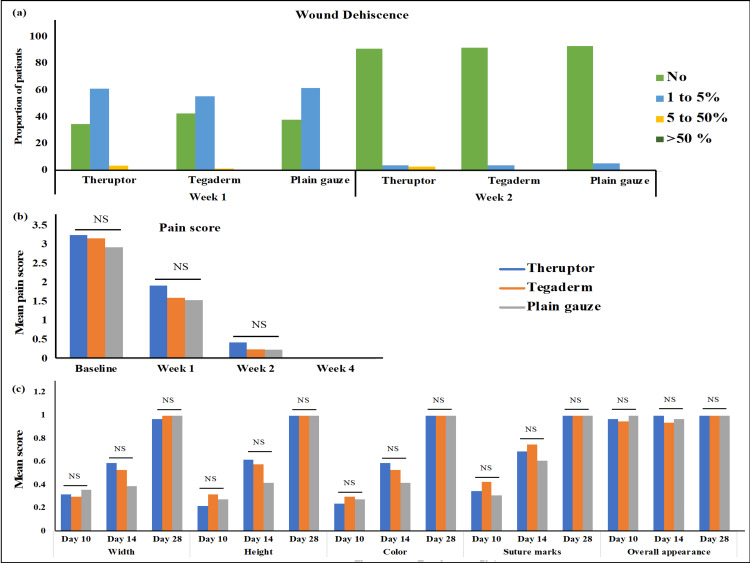
Wound dehiscence, wound pain assessment, and wound category among the groups. Bar graphs representing (a) the proportions of patients belonging to different groups with the differential percentages of wound dehiscence categorized as no (0%), 1-5%, 5-50%, and >50%; (b) the mean values of wound pain scorings at baseline and weeks 1, 2, and 4; and (c) the mean values of sub-categories of Stony Brook Scar Evaluation Scale at day 10, day 14, and day 28. NS: non-significant.

Wound dehiscence was categorized based on the percentage of opening on the wound from small opening to partial opening. It was categorized as the percentage of dehiscence as 0% (no wound dehiscence), 1-5%, 5-50%, and more than 50%, and the proportion of patients in these categories was estimated during follow-up visits of four weeks. On day 7, 52% of patients in the Tegaderm and 62% of patients each in the Theruptor and plain gauze arm had 1-5% of wound dehiscence; two patients in the Theruptor group and one patient in the Tegaderm group had wound dehiscence percentage of 5-50% on days 7 and 14. On day 14, 92% of patients in each intervention arm had no wound dehiscence, and three patients from each intervention arm had 1-5% of dehiscence. On days 21 and 28, all patients in all intervention groups showed no wound dehiscence except one patient belonging to the Tegaderm group who had wound dehiscence of >50% due to SSI. When the proportion of patients with differential percentages of wound dehiscence was compared, no significant difference was observed during the follow-up study (p > 0.05) (Figure 3, Panel a).

One patient in the Tegaderm group who had >50% dehiscence due to SSI received systemic antibiotics (cefixime) and surgical intervention in the form of re-suturing. However, no additional interventions were done for any of the other patients with wound dehiscence except for routine wound hygiene and dressing changes.

Wound Pain Score Assessment

At baseline and during the follow-up visits on days 7, 14, and 28, there was no statistically significant difference in the mean wound pain scores among the intervention groups (p > 0.05). The wound pain assessment data is shown in Figure 3, Panel b.

Clinical Assessment of Wounds

The wound was clinically assessed and graded for purulent drainage, hemorrhage, swelling, erythema, and scab on days 3 and 7. During the follow-up study, no patient had copious grades for any of the parameters. The majority of the patients were healed by day 7; therefore, the wounds were graded on days 3 and 7. More than 91% of patients belonging to each intervention arm had no purulent drainage, hemorrhage, or scab on days 3 and 7. It was noticed that more than 64% of all patients had no swelling and erythema while the wound was graded as scant in 30% of patients on day 3. The percentage of patients with no swelling and erythema increased to more than 92% on day 7. On day 3, when the three intervention groups were compared for scab formation, a significant difference was observed in the number of patients with scant-graded scab, belonging to the Theruptor and plain gauze dressing versus the Tegaderm group (1.6% and 1.5% vs. 9%; p < 0.05). One patient in the Tegaderm group had severe purulent drainage, hemorrhage, swelling, erythema, and scab because of SSI. Overall, there was no significant difference in the proportion of patients with differential wound grades (p > 0.05; Table 2).

**Table 2:** Clinical assessment of the wound during the study. n = number of patients; % = percentage

Parameters, n (%)	Theruptor (n = 62)	Tegaderm (n = 67)	Plain gauze (n = 68)	P-value
Day 3				
Purulent drainage				0.656
None	61 (98.4)	64 (95.5)	67 (98.5)	
Scant	1 (1.6)	2 (3)	1 (1.5)	
Moderate	-	1 (1.5)	-	
Hemorrhage				0.987
None	57 (92)	62 (92.5)	63 (92.6)	
Scant	5 (8)	5 (7.5)	5 (7.4)	
Swelling				0.214
None	40 (64.5)	44 (65.7)	54 (79.4)	
Scant	21 (33.9)	23 (34.3)	13 (19.1)	
Moderate	1 (1.6)	-	1 (1.5)	
Erythema				0.343
None	42 (67.7)	43 (64.2)	49 (72.1)	
Scant	20 (32.3)	22 (32.8)	19 (27.9)	
Moderate	-	2 (3)	-	
Scab				0.044
None	61 (98.4)	61 (91)	67 (98.5)	
Scant	1 (1.6)	6 (9)	1 (1.5)	
Day 7				
Purulent drainage				0.784
None	60 (97)	65 (97)	67 (98.5)	
Scant	2 (3)	2 (3)	1 (1.5)	
Hemorrhage:				0.377
None	62 (100)	66 (98.5)	68 (100)	
Scant	-	1 (1.5)	-	
Swelling				0.335
None	61 (98.4)	67 (100)	68 (100)	
Moderate	1 (1.6)	-	-	
Erythema				0.281
None	57 (92)	65 (97)	66 (97)	
Scant	5 (8)	2 (3)	2 (3)	
Scab:				0.397
None	60 (98.4)	64 (100)	63 (98.4)	
Scant	1 (1.6)	-	-	
Moderate	-	-	1 (1.6)	
Missing	1	3	4	

Product Performance Assessment

The mean values of all three dressings were compared for the product usage parameters. Theruptor and Tegaderm dressings were equally better and more efficacious than plain gauze dressing in terms of ease of application (82.87% and 84.13% vs. 71.7%), ease of removal (83.09% and 83.67% vs. 70.79%), comfort to wear (82.59% and 84.47% vs. 72.83%), exudate management (84.35% and 85.7% vs. 77.23%), mean wear time in hours (65.57 hours and 65.92 hours vs. 49 hours), and mobility of the patient (p < 0.05). The mobility of the patient was rated as normal, good, satisfied, and not satisfied. Overall, 20% of patients in the plain gauze dressing group were “not satisfied” compared to 1-3% of the patients in the Theruptor and Tegaderm groups. The data were found to be statistically significant (p < 0.05). However, there was no significant difference in the percentage of other parameters such as discoloration and pain on dressing application and removal among the intervention arms (p > 0.05). The product performance assessment data are summarized in Table 3.

**Table 3:** Product usage assessment. SD = standard deviation; n = number of patients; % = percentage; 0.0001 = Theruptor and Tegaderm showed better results than plain gauze dressing

Parameters (mean ± SD)	Theruptor (n = 62)	Tegaderm (n = 67)	Plain gauze (n = 68)	P-value
Ease of application	82.87 ± 16.19	84.13 ± 16.07	71.77 ± 19.88	0.0001*
Ease of removal	83.09 ± 14.19	83.67 ± 13.58	70.79 ± 19.64	0.0001*
Comfortable to wear	82.59 ± 18.94	84.47 ± 15.47	72.83 ± 20.88	0.0007*
Discoloration of the surrounding skin	8.21 ± 19.32	10.21 ± 21.24	10.5 ± 19.88	0.783
Pain on dressing application	9.52 ± 17.31	11.92 ± 20.32	15.7 ± 20.25	0.193
Pain on dressing removal	10.78 ± 19.73	12.51 ± 21.14	17.28 ± 23.07	0.203
Exudate and leakage handling	84.35 ± 20.47	85.7 ± 18.2	77.23 ± 19.61	0.030*
Mean wear time in hours	65.57 ± 5.2	65.92 ± 5.28	49 ± 8.31	0.0001*
Mobility of the patient, n (%)				0.0001*
Normal	26 (42)	27 (41)	35 (51)	
Good	20 (32)	26 (39)	11 (16)	
Satisfied	14 (23)	13 (19)	9 (13)	
Not satisfied	2 (3)	1 (01)	13 (20)	

Modified Hollander Wound Evaluation Scale

The cosmetic outcomes of the dressings were evaluated using the Modified Hollander Wound Evaluation scale. Among the three intervention arms, there was no significant difference in the mean scores of cosmetic outcomes on days 3 and 7 in the Theruptor, Tegaderm, and plain gauze dressing groups (0.08 ± 0.52 and 0.06 ± 0.51; 0 and 0; 0 and 0; p-values 0.218 and 0.328, respectively).

Stony Brook Scar Evaluation Scale Category

When all the sub-categories of SBSES, i.e., height, width, color, suture marks, and overall appearance were compared in all three intervention arms, no significant difference was observed in the mean values of categories during the follow-up evaluations on days 10, 14, and 28 (p > 0.05). The data are shown in Figure 3, Panel c.

Overall Quality of Life

During the follow-up periods, the overall quality of life improved in all three intervention arms. However, no significant difference was observed in the mean values of overall quality of life when all the groups were compared during the follow-ups on days 3, 7, 14, and 28 (p > 0.05) (Table 4).

**Table 4:** Wound impact evaluation and quality of life. n = number of patients; % = percentage

Parameters, n (%)	Theruptor (n = 62)	Tegaderm (n = 67)	Plain gauze (n = 68)	P-value
Overall quality of life				
Day 3	5.99 ± 2.16	6.36 ± 2.09	6.17 ± 2.08	0.582
Day 7	7.76 ± 2.01	8.02 ± 1.83	7.62 ± 2.02	0.51
Day 14	8.63 ± 2.37	9.14 ± 2.23	9.44 ± 1.5	0.323
Day 28	9.88 ± 0.32	10 ± 0	9.79 ± 0.79	0.314
Cardiff Wound Impact Questionnaire scores				
Day 3	3.42 ± 2.44	3.13 ± 1.53	3.66 ± 1.39	0.357
Day 7	1.67 ± 0.86	1.6 ± 0.99	2.09 ± 1.35	0.443
Social life (stressful)				
Day 3	3.32 ± 2.76	3.23 ± 1.67	3.92 ± 1.99	0.278
Day 7	2.01 ± 1.23	1.32 ± 1.08	2.07 ± 1.52	0.116
Social life (experience)				
Day 3	3.01 ± 1.96	2.99 ± 1.72	3.39 ± 1.75	0.364
Day 7	1.95 ± 1.01	1.96 ± 0.98	2.32 ± 1.45	0.439
Well-being				
Day 3	3.83 ± 2.19	3 ± 1.09	3.43 ± 1.34	0.3
Day 7	1.42 ± 0.87	1.91 ± 0.87	1.99 ± 1.65	0.404
Physical symptoms and daily living				
Day 3	3.13 ± 1.19	3.33 ± 1.63	3.89 ± 1.99	0.357
Day 7	1.29 ± 0.34	1.21 ± 1.02	1.98 ± 0.79	0.288

Cardiff Wound Impact Questionnaire

The scores of the Cardiff Wound Impact Questionnaire on days 3 and 7 are summarized in Table 4. When all three groups were compared, the mean scores of the Cardiff Wound Impact Questionnaire were found to be similar and comparable. The data were found to be non-significant (p > 0.05) during the follow-up evaluations.

Cost of Wound Management With the Dressing

The cost of wound management involved with each dressing was calculated by estimating the average unit price of the dressing, the number of dressing changes required, and the expenses involved for personnel and consumables. It was found that the number of dressing changes required was significantly higher in plain gauze dressing compared to Theruptor and Tegaderm dressings (p < 0.0001). Although the average unit price of plain gauze was very low compared to other dressings, the expenses involved for personnel and consumables were comparatively higher. Overall, the total cost of Theruptor dressing was significantly less than Tegaderm and plain gauze dressings (p < 0.0001) (Table 5).

**Table 5:** Cost of wound management with the dressing. The cost is mentioned in rupees (₹). SD = Standard deviation; n = number of patients

Parameters (mean ± SD)	Theruptor (n = 62)	Tegaderm (n = 67)	Plain gauze (n = 68)	P-value
Number of dressing changes required	2.28 ± 0.5	2.2 ± 0.35	2.83 ± 0.22	0.0001
Average unit price (₹)	140	320	73.80	
Dressing costs	319.2 ± 77.1	704 ± 217.62	208.85 ± 51.61	0.0001
Personnel involved and consumables	798 ± 192.76	770 ± 238.02	990.5 ± 244.76	0.0001
Total cost	1,117.2 ± 269.86	1,474 ± 455.63	1,199.3 ± 296.36	0.0001

Complications After Surgery

Based on the Clavien-Dindo classification, the complications after surgery were classified in the intention-to-treat population of 210 patients. Among them, only one patient belonging to the Tegaderm group reported complications post-surgery and was classified under grade III of the Clavien-Dindo classification. The patient underwent umbilical hernioplasty and developed SSI, which required further surgical re-intervention.

Adverse Events

In a total of 210 patients, AEs were observed in three patients, two patients in the Theruptor group (one developed blister and one developed itching in the surgical site) and one patient in the Tegaderm group who developed SSI and required further surgical procedure.

## Discussion

Wound healing is a multifactorial process that involves crosstalk between immune cells and the surrounding environment. Poor wound management affects millions of people every year [21]. Therefore, the selection of appropriate wound dressing is imperative for effective wound healing. An ideal wound dressing should have unique properties such as a moist wound healing environment, optimum exudate management, pain reduction, comfort during usage and removal, and cost-effectiveness [14]. To our knowledge, our study is one of its kind to provide a comparative assessment of clinical efficacy and safety of three wound dressings, i.e., Theruptor, Tegaderm, and plain gauze, in patients undergoing abdominal and joint surgeries.

In this study, the rate of wound healing capability of all three dressings was assessed, and it was found that more than 92% of patients were healed on day 7. In a prospective randomized study, Ravenscroft et al. (2006) compared the wound healing capacity of cutiplast and Tegaderm in hip and knee surgery. The study found that the wound healing capacity of Tegaderm is approximately 82%, which is a little less than our observed data [21]. Further, the median healing time was estimated to be seven days in our study. In a study, Ong et al. (2015) evaluated the performance and safety of Tegaderm dressings in the porcine wound healing model and observed the average rate of wound closure ranged from 7 to 21 days, which is in concordance with our data [22]. Several studies have reported that SSIs lead to prolonged hospital stays, increased cost, amputation, re-surgeries, and even mortality. Regarding SSI, all three dressings showed similar efficacy in reducing the microbial load during different days of postoperative care. It was noted that the incidence of SSI decreases in a time-dependent manner with all three interventions from 17% on day 0 to 1% on day 10. In our previous in-vitro study, Theruptor showed antimicrobial properties with microbial reduction of >5 log at one hour till 28 days [14]. In a retrospective study, Fakhoury et al. (2019) reviewed Dermabond and Tegaderm as better incision dressing in patients undergoing vascular surgeries and found the SSI rate ranging from 3.7 to 16% [23]. The data are in concordance with our study. Overall, all three dressings were equally effective in healing the wound and reducing the incidence of SSI.

Next, the wound dehiscence was assessed and compared in all three intervention groups. Most wounds were healed on day 7 and no dehiscence was observed in the majority of the patients belonging to the differential intervention groups except for one patient in the Tegaderm group. A study conducted by Fakhoury et al. (2019) reported zero wound dehiscence with Dermabond-Tegaderm combination in patients undergoing vascular surgery, which supports our findings [23]. Further, the wound pain was found to be reduced with time in all three intervention groups during the follow-up evaluations. All three interventions had similar wound pain scores. In a dissertation study, Premlatha (2014) assessed and compared the effectiveness of Tegaderm and Dynaplaster in 30 children. Regarding pain, the authors observed “no pain” in 63.3% of children with a pain score of 1.06 ± 0.09 in the Tegaderm group [24]. A study conducted by Ravenscroft et al. (2008) reported the mean wound pain scores in the Tegaderm group ranging from 0.8 to 1.6 [20]. The data of both these studies are in concordance with our results, suggesting the efficacy of Tegaderm in reducing wound dehiscence and pain which was found to be similar in the Theruptor and plain gauze dressing groups.

SBSES consists of five domains, namely, width, height, color of the scar, presence of suture marks, and overall appearance [18]. All three interventions performed equally well in improving the cosmetic outlook of the wounds with time during the follow-up period. Regarding product performance, we found a significant and “good” product performance of Theruptor and Tegaderm in comparison to plain gauze dressing in our study. In a prospective randomized clinical trial, Brown-Etris et al. (2008) found Tegaderm’s performance to be better than hydrocolloid dressing in terms of comfort, dressing performance, and wound healing in the management of pressure ulcers [25] which supports our data.

Then, the overall cost of the dressing was estimated through the number of dressing changes required, average unit price, and expenses involved for personnel and consumables. It was estimated that the total cost of wound management in the case of Theruptor dressing was significantly lesser than Tegaderm (30%) and plain gauze dressings (7%) (p < 0.0001), suggesting Theruptor as a market equivalent and an alternative of Tegaderm.

This study had a few limitations: Patients with joint surgeries were very few in the study, and the decision of dressing change was solely taken by the staff depending on their clinical judgment.

## Conclusions

The primary and secondary outcome data provide substantial evidence of the clinical safety and efficacy of Theruptor dressing which is equivalent to Tegaderm dressing. However, the product performance of Theruptor and Tegaderm dressings was significantly better than plain gauze dressing. In addition, Theruptor is cost-effective and may replace Tegaderm and plain gauze dressing with similar properties of wound healing rate, safety, and product performance. Conclusively, Theruptor may be considered a dressing of choice in the postoperative wound management of abdominal and joint surgeries where effective and faster healing is required.

## References

[1] Haque M, Sartelli M, McKimm J, Abu Bakar M (2018). Health care-associated infections - an overview. Infect Drug Resist.

[2] Ducel G, Fabry J, Nicolle L (2002). Prevention of Hospital-Acquired Infections: A Practical Guide. Geneva.

[3] Klevens RM, Edwards JR, Richards CL Jr, Horan TC, Gaynes RP, Pollock DA, Cardo DM (2007). Estimating health care-associated infections and deaths in U.S. hospitals, 2002. Public Health Rep.

[4] Monegro AF, Muppidi V, Regunath H (2023). Hospital-Acquired Infections. https://www.ncbi.nlm.nih.gov/books/NBK441857/.

[5] Nilsson L, Risberg MB, Montgomery A, Sjödahl R, Schildmeijer K, Rutberg H (2016). Preventable adverse events in surgical care in Sweden: a nationwide review of patient notes. Medicine (Baltimore).

[6] Hassan RS, Osman SO, Aabdeen MA, Mohamed WE, Hassan RS, Mohamed SO (2020). Incidence and root causes of surgical site infections after gastrointestinal surgery at a public teaching hospital in Sudan. Patient Saf Surg.

[7] Nuvials X, Palomar M, Alvarez-Lerma F (2015). Health-care associated infections. Patient characteristics and influence on the clinical outcome of patients admitted to ICU. Envin-Helics registry data. Intensive Care Med Exp.

[8] Watanabe A, Kohnoe S, Shimabukuro R (2008). Risk factors associated with surgical site infection in upper and lower gastrointestinal surgery. Surg Today.

[9] Seidelman JL, Mantyh CR, Anderson DJ (2023). Surgical site infection prevention: a review. JAMA.

[10] Sood A, Granick MS, Tomaselli NL (2014). Wound dressings and comparative effectiveness data. Adv Wound Care (New Rochelle).

[11] Westby MJ, Dumville JC, Soares MO, Stubbs N, Norman G (2017). Dressings and topical agents for treating pressure ulcers. Cochrane Database Syst Rev.

[12] Kerr AJ, Arrowsmith M (2014). An evaluation of 3M Tegaderm Superabsorber dressing using an exudate management algorithm. Br J Community Nurs.

[13] Michelin RM, Ahdoot E, Zakhary BL, McDowell M, French M (2021). Choosing the optimal wound dressing for bathing after total knee arthroplasty. J Arthroplasty.

[14] Gupta R, Murthy KVNNS, Bhagavan KR, Moharana AK, Rodrigues M, TS D (2022). Antimicrobial properties of Theruptor 3D-hydrocellular wound dressing: an in vitro study. Int J Surg Open.

[15] Schulz KF, Altman DG, Moher D (2010). CONSORT 2010 statement: updated guidelines for reporting parallel group randomised trials. BMJ.

[16] Morand GB, Anderegg N, Kleinjung T (2021). Assessment of surgical complications with respect to the surgical indication: proposal for a novel index. Front Surg.

[17] Ademuyiwa AO, Sowande OA, Adejuyigbe O, Usang UE, Bakare TI, Anyanwu LJ (2009). Evaluation of cosmetic appearance of herniotomy wound scars in African children: comparison of tissue glue and subcuticular suturing. Indian J Plast Surg.

[18] Fearmonti R, Bond J, Erdmann D, Levinson H (2010). A review of scar scales and scar measuring devices. Eplasty.

[19] Price P, Harding K (2004). Cardiff Wound Impact Schedule: the development of a condition-specific questionnaire to assess health-related quality of life in patients with chronic wounds of the lower limb. Int Wound J.

[20] Ravenscroft MJ, Harker J, Buch KA (2006). A prospective, randomised, controlled trial comparing wound dressings used in hip and knee surgery: Aquacel and Tegaderm versus Cutiplast. Ann R Coll Surg Engl.

[21] Han G, Ceilley R (2017). Chronic wound healing: a review of current management and treatments. Adv Ther.

[22] Ong CT, Zhang Y, Lim R (2015). Preclinical evaluation of Tegaderm™ supported nanofibrous wound matrix dressing on porcine wound healing model. Adv Wound Care (New Rochelle).

[23] Fakhoury E, Lau I, Finlay DJ (2019). Dermabond and Tegaderm: a better surgical incision dressing. Ann Vasc Surg.

[24] (2023). Effectiveness of Tegaderm versus Dynaplaster upon pain perception and occurence of infection during removal among children. http://repository-tnmgrmu.ac.in/349/1/300212814premalatha.pdf.

[25] Brown-Etris M, Milne C, Orsted H (2008). A prospective, randomized, multisite clinical evaluation of a transparent absorbent acrylic dressing and a hydrocolloid dressing in the management of Stage II and shallow Stage III pressure ulcers. Adv Skin Wound Care.

